# IL-6 During Influenza-*Streptococcus pneumoniae* Co-Infected Pneumonia—A Protector

**DOI:** 10.3389/fimmu.2019.03102

**Published:** 2020-01-21

**Authors:** Xuemei Gou, Jun Yuan, Hong Wang, Xiaofang Wang, Jiangming Xiao, Jingyi Chen, Shuang Liu, Yibing Yin, Xuemei Zhang

**Affiliations:** ^1^Key Laboratory of Diagnostic Medicine Designated by the Ministry of Education, Chongqing Medical University, Chongqing, China; ^2^Department of Laboratory Medicine, The Children's Hospital of Chongqing Medical University, Chongqing, China

**Keywords:** influenza, *Streptococcus pneumoniae*, pneumonia, IL-6, co-infection

## Abstract

Understanding of pathogenesis and protection mechanisms underlying influenza-*Streptococcus* pneumoniae co-infection may provide potential strategies for decreasing its high morbidity and mortality. Interleukin-6 (IL-6) is an important cytokine that acts to limit infection-related inflammation; however, its role in co-infected pneumonia remains unclear. Here we show that the clinically relevant co-infected mice displayed dramatically elevated IL-6 levels; which was also observed in patients with co-infected pneumonia. *IL-6*^−/−^ mice presented with increased bacterial burden, early dissemination of bacteria to extrapulmonary sites accompanied by aggravated pulmonary lesions and high mortality when co-infection. This protective function of IL-6 is associated with cellular death and macrophage function. Importantly, therapeutic administration of recombinant IL-6 protein reduced cells death in BALF, and enhanced macrophage phagocytosis through increased MARCO expression. This protective immune mechanism furthers our understanding of the potential impact of immune components during infection and provides potential therapeutic avenues for influenza-*Streptococcus pneumoniae* co-infected pneumonia.

## Introduction

Influenza A virus (IAV) and *Streptococcus pneumoniae* (*S. pneumoniae*) are two of the major causes of respiratory infections in humans. Interestingly, more than 95% of all severe illnesses and deaths in the 1918 and 2009 influenza pandemics (H1N1 pandemics) were complicated by bacterial co-infection, most of these (~50%) were *S. pneumoniae* infection (1–4). Thus, influenza-associated mortality is partially the result of complications arising from secondary bacterial infection. In addition to *S. pneumoniae, Staphylococcus aureus, H. influenzae* and other often colonized bacteria are identified in secondary infections following infection with influenza (3). Co-infections with IAV and bacteria are a global health concern. In the 1918 influenza pandemic, more than 50 million individuals died from pneumonia exacerbated by bacterial superinfection (1, 4). This death toll was higher than the total number of deaths in World War I. Supporting this concern numerous studies in co-infected pneumonia mouse models have reported complex interactions between virus, bacteria and host, leading to failed barrier function in the lungs (5), dysregulated immune responses (2, 6, 7) and unbalanced homeostasis (3). Although past studies have started to explore the mechanisms underlying influenza-induced morbidity and mortality, they remain poorly understood, and thus a better understanding of secondary infection is a priority.

IL-6 is a commonly detected cytokine and one of the most induced immune markers during co-infection (8, 9); however, its role in these infections has not been defined. IL-6 (26 kDa in size) is a four-helical cytokine consisting of 184 amino acids, encoded by the *IL6* gene (10). The IL-6 can be secreted in many cell types upon appropriate stimulation during infection, inflammation or cancer (11). Over the past 30 years, a wealth of data generated from *ex vivo* and *in vivo* investigations have supported IL-6 is a pleiotropic cytokine and a major player in integrated immunity via its role in defense against viral (12–14), parasitic (15), fungal (16) and bacterial infections (17, 18). In studies focused on IL-6 and *S. pneumoniae* infection, IL-6 levels were elevated and these elevated IL-6 were protective to the host defending against pneumococcal pneumonia by down-regulating the activation of the cytokine network (18, 19). However, the detail of this protective mechanism is still unclear. IL-6 has been shown to be important during influenza pathogenesis in the lungs. Studies in *IL-6*^−/−^ mice showed reduced disease resistance in these animals with changes in various protective mechanisms including loss of balance in T-cell responses and inflammatory resolution (13, 20), as well as prolonged neutrophil survival (14). In addition, elevated IL-6 during influenza infection has been associated with haemophagocytic syndrome resulting increased lung damage and worse outcome (21). In short, IL-6 levels vary widely depending on the type, severity and location of disease, and may result in either protection from or exacerbation of disease (11, 22).

Previous reports have shown that IL-6 was detected in the lungs and circulation of patients from the 1918 and 2009 H1N1 influenza pandemics (1, 23–25). IL-6 as the most upregulated cytokine upon IAV-*S. pneumoniae* co-infected mice have been revealed in recent researches (8, 9). Our studies also found that IL-6 concentration is significantly higher in co-infection of IAV-*S. pneumoniae* than in infection with either pathogen alone. However, little is known about the role of IL-6 in co-infected pneumonia. Thus, here we sought to clear if IL-6 contributes to lung pathology or physiology during co-infection.

Here, we evaluated the immune response to IAV-*S. pneumoniae* co-infection at different time points and found evidence, from both animal models and partial clinical data, for protective functions of IL-6 during co-infections. IL-6 has been shown to decrease susceptibility to co-infection by reducing cells death following IAV-triggered death in the lungs. In addition, IL-6 has been shown to improve bacterial phagocytosis by up-regulating MARCO surface expression in macrophages during secondary bacterial infection. These results show that IL-6 is an important determinant in controlling bacterial invasion and dissemination. These assign IL-6 an unexpected homeostatic role in limiting infection-related inflammation. This protective immune mechanism will help researchers understand the potential impact of immune components and provide potential therapeutic options for IAV-*S. pneumoniae* co-infected pneumonia.

## Patients and Methods

### Human Studies

Child patients with co-infected pneumonia, influenza pneumonia and pneumococcal pneumonia, were recruited from the Children's Hospital of Chongqing Medical University. Diagnosis of pneumonia was based on respiratory symptoms with at least one of the following: fever (temperature ≥38.0°C) or hypothermia (temperature <35.0°C), runny rose, sneezing, cough, shortness of breath, pleuritic chest pain, and altered breath sounds on auscultation, according to WHO guidelines (2016). An individual with laboratory-confirmed IAV infection confirmed by one or more of the following tests: polymerase chain reaction (PCR) or 4-fold rise in virus-specific neutralizing antibodies. An individual with positive pneumococcal culture result was considered *S. pneumoniae* infection. Both positive IAV and *S. pneumoniae* results were thought co-infections. Patients undergoing organ transplantation, those with immunodeficiency, or receiving immunosuppressive agents in the previous 8 weeks were excluded from the study. Control serum samples were obtained from 45 physical examination healthy donors. This study was carried out in accordance with the recommendations of the Clinical Research Ethics Committee of the Children's Hospital of Chongqing Medical University. The protocol was approved by the Clinical Research Ethics Committee of the Children's Hospital of Chongqing Medical University. All subjects gave written informed consent in accordance with the Declaration of Helsinki.

### Mice

All mice used in this study were 8–10 weeks old C57BL/6 mice of both genders. In all experiments, control and experimental mice were obtained from and bred in Chongqing Medical University. *IL-6*^−/−^ mice were purchased from The Jackson Laboratory (Bar Harbor, ME). All mice were housed under humidity- and temperature- controlled specific pathogen-free conditions in the animal facility of Chongqing Medical University. This study was carried out in accordance with the principles of the Basel Declaration and recommendations of the guidelines of the Institutional Animal Care and Use Committee of the Chongqing Medical University. The protocol was approved by the Institutional Animal Care and Use Committee of the Chongqing Medical University.

### Infectious Reagents

Influenza virus strain A/Puerto Rico/8/1934 (ATCC-VR-1469, along to H1N1) was replicated within the allantoic cavities of 9-day-old specific pathogen-free embryonated chicken eggs by viral injection. The allantoic fluid was then harvested, clarified by centrifugation at 680 g for 10 min. Supernatants were stored in aliquots at −80°C. The viruses were quantified in Madin Darby Canine Kidney (MDCK) cells and expressed as PFUs. *S. pneumoniae* D39 (ATCC-6302) was stored at −80° and grown in C + Y (casamino acid & yeast extract) medium at 37°C with 5% CO_2_ to an optical density of 0.45–0.5, then collected by centrifugation and re-suspended in PBS immediately prior to infection.

### IAV-*S. pneumoniae* Co-Infected Pneumonia Model

Mice were anesthetized by intraperitoneal injection with 1.5% pentobarbital sodium (0.1 ml/20 g of body weight) and infected intranasally with a dose of 400 PFUs of IAV in 30 μl (15 μl per nostril) sterile phosphate-buffered saline (PBS) or control PBS alone. At specific time points following IAV infection, the mice were treated by intranasal injection with 1 × 10^8^ CFUs of D39 suspended in 30 μl PBS or control PBS. This progress mimicked the natural route of pneumococcal infection. Day 0 was indicated as viral infection; days post IAV infection were indicated as number outside parenthesis and the number in parenthesis indicated days post *S. pneumoniae* infection, such as 6 dpi (1 dpi).

### Survival, Body Weights and Determination of Bacterial and Viral Burdens

Mice were weighed and monitored for signs of morbidity and mortality daily. Bacterial burden (CFU) was determined by plating titrating doses of organ homogenate on agar plates as previously described (26). Viral titers (PFUs) in the lungs were determined by titration of serial 10-fold dilutions of the lung homogenates on MDCK cells as previously described (24). Viral RNA copies per lung were determined by levels of PR8 *M1*gene expression by RT-PCR. Primers for influenza matrix *M1* gene were as follows: forward: 5′-TGAGTCTTCTAACCGAGGTC-3′; reverse: 5′-GGTCTTGTCTTTAGCCATTCC-3′. The number of copies of the *M1* gene was calculated using an M1-containing plasmid of known concentration as a standard (26).

### Histology and *In Situ* Cell Death Detection of Lung Tissue

Whole lungs were perfused with 500 μl of 4% neutral paraformaldehyde *in situ* and then fixed for 24 h in 4% neutral paraformaldehyde. Five-micrometer sections from fixed, paraffin-embedded whole lung were stained using hematoxylin-and-eosin and examined by light microscopy. The scoring system was based on one that was previously report (27), briefly: 0 = no injury (apparently normal tissue); 1 = discrete and slight, when the lesion occupies <25% of the tissue; 2 = moderate when the lesion occupies 25–50% of the tissue; and 3 = intense, a severe injury with diffuse or focal inflammation around all structure of biopsy occupying more than 50% of the tissue.

Detection and quantification of apoptosis in lung tissues were performed on paraffin embedded tissue sections using the *in situ* cell death detection kit (Fluorescein, Roche) according to the manufacturer's protocol. The fluorescent images were analyzed using a fluorescence microscope (Nikon ECLIPSE 80i, Japan).

### Wet to Dry Ratio of Lung

The wet/dry ratio of lung samples was calculated from the initial weight of the whole lung (wet weight) to its weight after desiccation at 70°C for 72 h (dry weight).

### Measurement of Cytokines and Chemokine

Inflammatory cytokines and chemokine, including IL-6, IL-1β, IL-10, IFN-γ, TNF-α, KC, CCL2, and IP-10 levels were measured by using a human or mouse enzyme-linked immunosorbent assay (ELISA) kit from Biolegend according to the manufacturer's protocol.

### Flow Cytometry Assays

The bronchoalveolar lavage fluid (BALF) was collected from the airways of experimental mice and then centrifuged. The cell pellet was resuspended in BD Red Blood Cell Lysis solution (as per the manufacturer's instructions) to remove Red blood cells. Following collection the cell pellet was immediately resuspended in 1 ml of PBS for counting. To analyze the cells types, cells were blocked with purified anti-mouse CD16/CD32 (BD Biosciences) and subsequently stained for flow cytometry as follows: APC-conjugated anti-CD11b (BD Biosciences), FITC-conjugated anti-Ly-6G (BD Biosciences), PE-cy5- conjugated anti-F4/80 (BD Biosciences). To analyze cellular apoptosis, BALF cells were stained with both FITC Annexin V and PI (BD Biosciences) in accordance with the manufacturer's instructions. To analyze the expression of MARCO, BALF cells were stained with both APC-conjugated anti-CD11b and Fluorescein-conjugated anti-MARCO (R&D). The stained cells were analyzed using BD FACSCalibur™ and FlowJo software.

### Isolation of Macrophages and Neutrophils

Peritoneal macrophages were induced by intraperitoneal injection of 1 ml of sterile liquid paraffin. After 5 days, macrophages were collected through peritoneal lavage with 5 ml cold PBS. Alveolar macrophages were collected by inflating five times with 1 ml cold PBS via an intratracheal cannula and was repeated a further two times. Macrophages were isolated by adherence 40 min on culture plastic. Neutrophils were purified from the bone marrow from mouse limbs. A cell suspension was mashed through a 70-lm filter, and neutrophils were separated from the single-cell suspension by positive selection on a MACS column using anti-Ly6G-biotin and anti-biotin microbeads (Miltenyi Biotec), as per the manufacturer's instructions.

### Phagocytosis Assay

For the *in vitro* phagocytosis assays, peritoneal macrophages from WT and *IL-6*^−/−^ mice were plated at 2 × 10^5^ cells/well in 24-well plates and allowed to adhere overnight. After washing, cells were treated with IAV and culture medium for 2 h, then infected with 2 × 10^7^ CFUs of *S. pneumoniae* for a multiplicity of infection (MOI) of 100:1 and cultured for 1 h to allow phagocytosis to occur. The plates were washed three times with PBS. Gentamicin (200 μg/ml) and penicillin (10 μg/ml) were added and then incubated for 15 min to kill free and extracellular adherent bacteria. Cells were then lysed and the extent of lysis was determined using a standard inverted microscope. CFUs were counted by plating undiluted and 10-fold diluted lysates on blood agar plates.

For the *in vivo* phagocytosis assays, co-infected WT and *IL-6*^−/−^ mice were established using 1 × 10^8^ CFUs of FITC-labeled heat-killed *S. pneumoniae* D39 as opposed to the previously described live D39. BALF was collected 4 h later; cells were washed and resuspended in PBS. To visualize cell nuclei, cells were stained with 10 μg/ml DAPI (4′, 6-diamidino−2-phenylindole). The fluorescent images were then observed and analyzed using a fluorescence microscope.

### mrIL-6 and Anti-IFN-γ Treatment

Mice were treated intranasally with 10 μg/30 μl/mouse recombinant mouse IL-6 protein (R&D) at the same time as the animals were challenged with *S. pneumoniae*. Mice were treated intranasally with 50 μg/20 μl/mice of anti-IFN-γ mAb (eBioscience) twice, once 1 day prior to *S. pneumoniae* challenge and the second at the time of challenge.

For the *in vitro* assays, macrophages were treated with 20 ng/ml of recombinant mouse IL-6 protein (R&D).

### Statistical Analysis

For survival studies, significance was assessed using the log-rank (Mantel–Cox) test. For other data (pneumococcal loads, cells and cytokines), statistical significance was determined using the Mann–Whitney U-test (two comparisons) or Kruskal-Wallis test (multigroup comparisons). All statistical comparisons were performed using Prism 7 (GraphPad Software, Inc.). A *p* < 0.05 was considered statistically significant.

## Results

### Primary Influenza Infection Increases the Susceptibility of WT Mice to Secondary *S. pneumoniae* Infection

**Figure 1:**
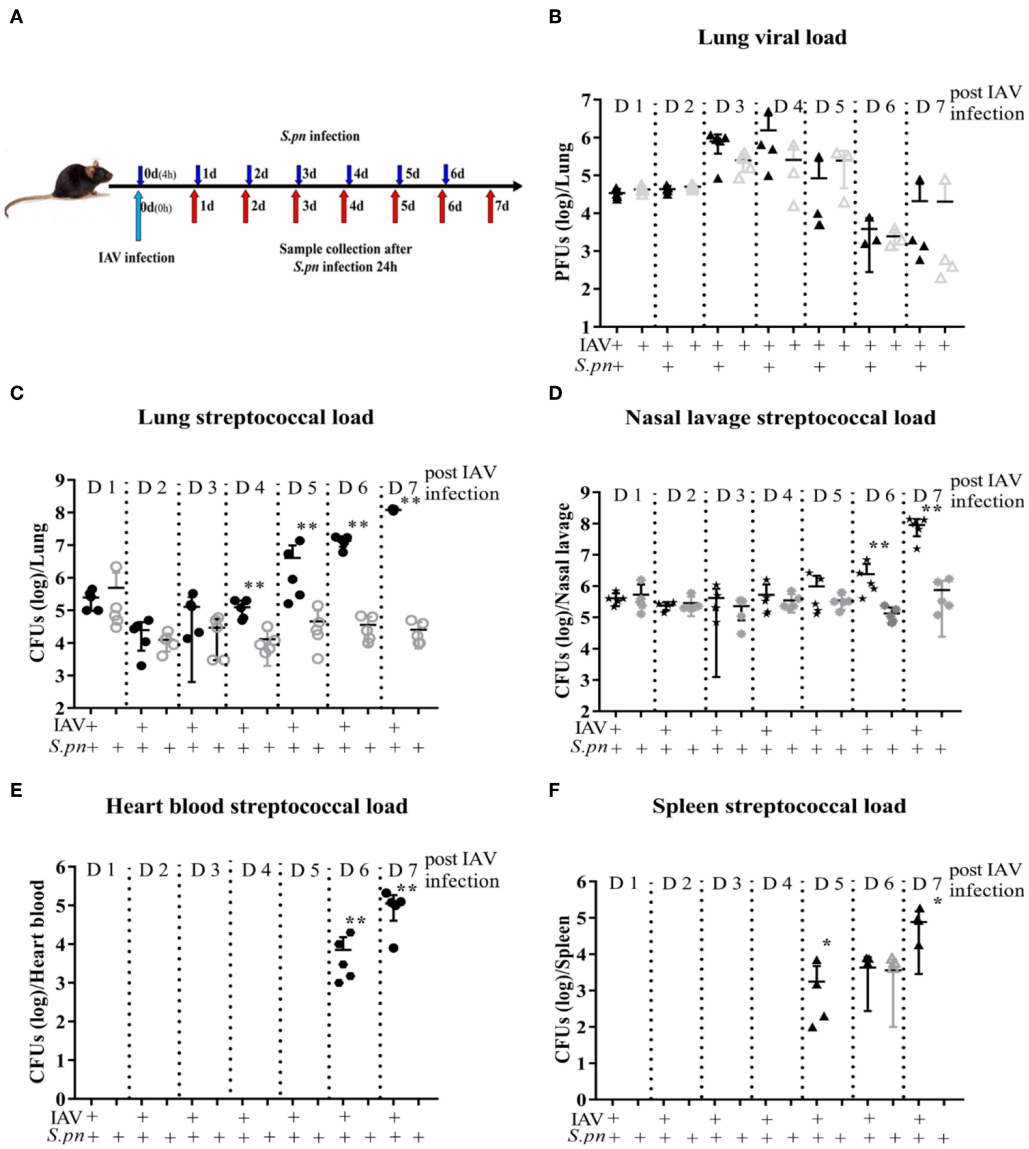
Primary influenza infection increases susceptibility to secondary *S. pneumoniae* infection in WT mice 4 days post IAV infection. **(A–F)** Co-infected WT mice were intranasally infected with 400 PFU/30 μl PR8 on day 0 and then challenged with 1 × 10^8^ CFU/30 μl of *S. pneumoniae* D39 4 h, day 1, day 2, day 3, day 4, day 5, and day 6 post IAV infection, respectively. Lungs were harvested 24 h after the final challenge. Single IAV-infected control mice were infected with 400 PFU/30 μl PR8 on day 0 and then were challenged with PBS/30 μl at the same time with *S. pneumoniae* infection. Lungs were harvested 24 h after the final challenge. Single D39-infected control mice were infected with PBS/30 μl on day 0 and then were challenged with 1 × 10^8^ CFU/30 μl of *S. pneumoniae* D39 as described above. Lungs were harvested 24 h after the final challenge. **(B)** Lung viral load was measured first using PFUs, *n* = 3–5/group. Streptococcal burden in the lung **(C)**, nasal lavage **(D)**, heart blood **(E)**, and spleen **(F)** were determined using CFUs following D39 challenge, *n* = 4–5/group. ^*^P < 0.05 and ^**^P < 0.001.

**Figure 2:**
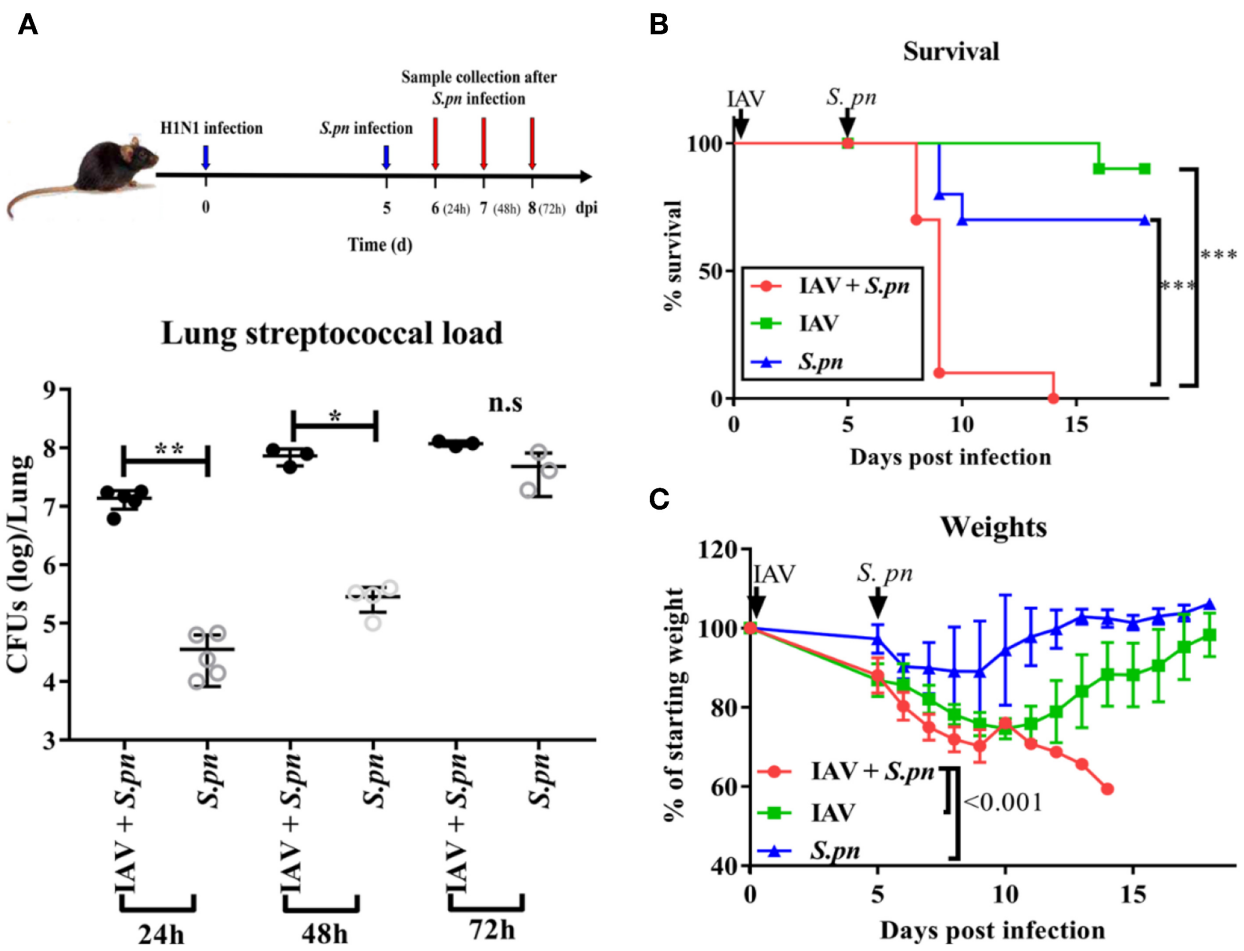
The survival of WT mice during severe co-infection. **(A)** Pneumococcal loads in the lung were pooled 24, 48, and 72 h after co-infection, *n* = 3–5/group. Mortality **(B)** and weight **(C)** following intranasal infection of C57BL/6 mice with IAV and *S. pneumoniae* [6 dpi (1dpi)], *n* = 10/group. ^*^P < 0.05, ^**^P <0.01, and ^***^P < 0.001.

A clinically relevant mouse model, was established as previously described (2). This model was used to mimic IAV-*S. pneumoniae* co-infected pneumonia in humans. To identify the kinetics of the nature, strength and timing of interactions between IAV and *S. pneumonia*, secondary *S. pneumoniae* infection was established by administration of the bacteria at day 0 (4 h), 1, 2, 3, 4, 5, and 6 post IAV infection (Figure 1A). The supernatants from lung homogenates harvested at 24 h post *S. pneumonia* infection, were then analyzed. No difference was found in lung viral titers between co-infection and IAV single infection (Figure 1B). The susceptibility of mice to secondary pneumococcal pneumonia was increased at 4 days post-IAV infection and peaked at 5–7 days (Figure 1C). After 6 days IAV infection, the nasal pharyngeal tissues, blood and spleen bacterial load in co-infected mice were significantly higher than during bacterial infection alone (Figures 1D–F). Our severe infected model implied that co-infected pneumonia progressed to sepsis post 6 days IAV infection. To further research the progression of co-infection, pneumococcal loads were profiled on days 6–8 (Figure 2A). Both single bacterial infection and co-infection, exhibited a gradual increase in pneumococcal loads as infection continued. However, the most significant difference in bacterial load were seen at 24 h after co-infection when compared with single bacterial infection at the same time (Figure 2A), indicating an important demarcation point for disease development. This led us to identify 6 dpi (1 dpi) (as previously described about method of model) as our mouse model for our later experiments.

After understanding the morbidity of our co-infection model, we observed the mortality. Co-infected mice displayed 100% mortality (Figure 2B). They had peak weight losses of 40% (Figure 2C). However, both single infected mice had less 30% mortality and weight loss. In agreement with the high mortality, the macroscopic pathological features of the lungs in co-infected animals were more obviously hemorrhagic and thrombotic syndrome, as indicated by the arrow (Figure S1A). The indicator of the lung edema—W/D ratio in the co-infected group was also higher than other groups (Figure S1B). IFN-γ, IL-1β, and TNF-α are important cytokines related to infection and immunity. Therefore, we profiled the response of these cytokines in our mouse model. These three cytokines were significantly increased during co-infection (Figure S1C).

To summarize, the initial influenza infection increases the susceptibility of bacterial infection after 4 days IAV infection, and bacterial infection spreads from the nasopharynx to the whole body as the infection continues.

### The Level of IL-6 Was Elevated in Influenza-*S. pneumoniae* Co-Infected Pneumonia

**Figure 3:**
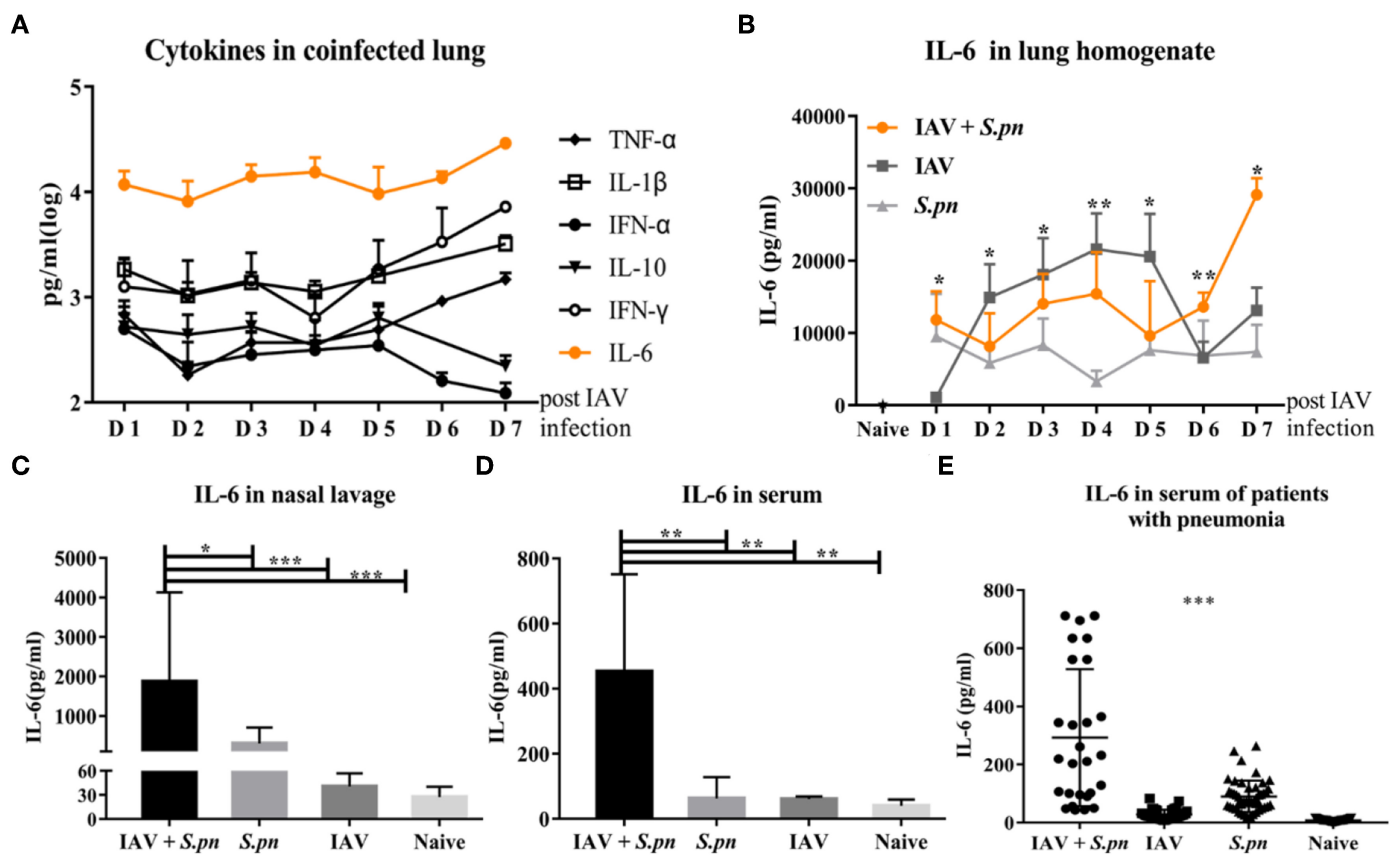
The level of IL-6 was elevated in influenza-*S. pneumoniae* co-infection. **(A)** Co-infected WT mice were established as described in Figure 1A. The dynamic changes of various cytokines in the lungs of co-infected mice at different time points, *n* = 5/group. **(B)** The co-infection and single infection were established as described in Figure 1A. The dynamic changes of IL-6 in the lungs of mice during co-infection and single infection at different time points, *n* = 5/group. **(C–D)** IL-6 levels in nasal lavage **(C)** and serum **(D)** of co-infected WT mice [6 dpi (1 dpi)], *n* = 5–10/group. **(E)** The levels of IL-6 in serum samples from patients with co-infected, viral, or bacterial pneumonia and healthy control groups, *n* = 27–50/group. ^*^P < 0.05, ^**^P < 0.01, and ^***^P < 0.001.

Next, we wanted to observe the differences in cytokines induced during co-infected lungs. To our surprise, IL-6 was the most prominent among all nine cytokines measured during co-infection (Figure 3A). We also compared the dynamic changes of IL-6 in co-infection and single infection. IL-6 concentration in co-infection was not always higher than in single infection, but it increased as prolonged infection time (Figure 3B). IL-6 production in nasal lavage fluid and serum of the co-infected group [6 dpi (1 dpi)] increased when compared with other groups (Figures 3C,D). To validate our findings, we measured IL-6 levels in the serum of patients with different pneumonia and healthy controls. Consistent with change of IL-6 in mice serum, we observed a significant increase in IL-6 production in serum of patients with co-infected pneumonia in Figure 3E. No correlation was observed between serum IL-6 levels and any other clinical features and no apparent difference in serum IL-6 between acute infection and recovery (data not shown).

### IL-6 Deficiency Obviously Increased the Predisposition to Secondary Bacterial Infection Post-influenza Infection and Decreased the Bacterial Clearance During Secondary Bacterial Infections

**Figure 4:**
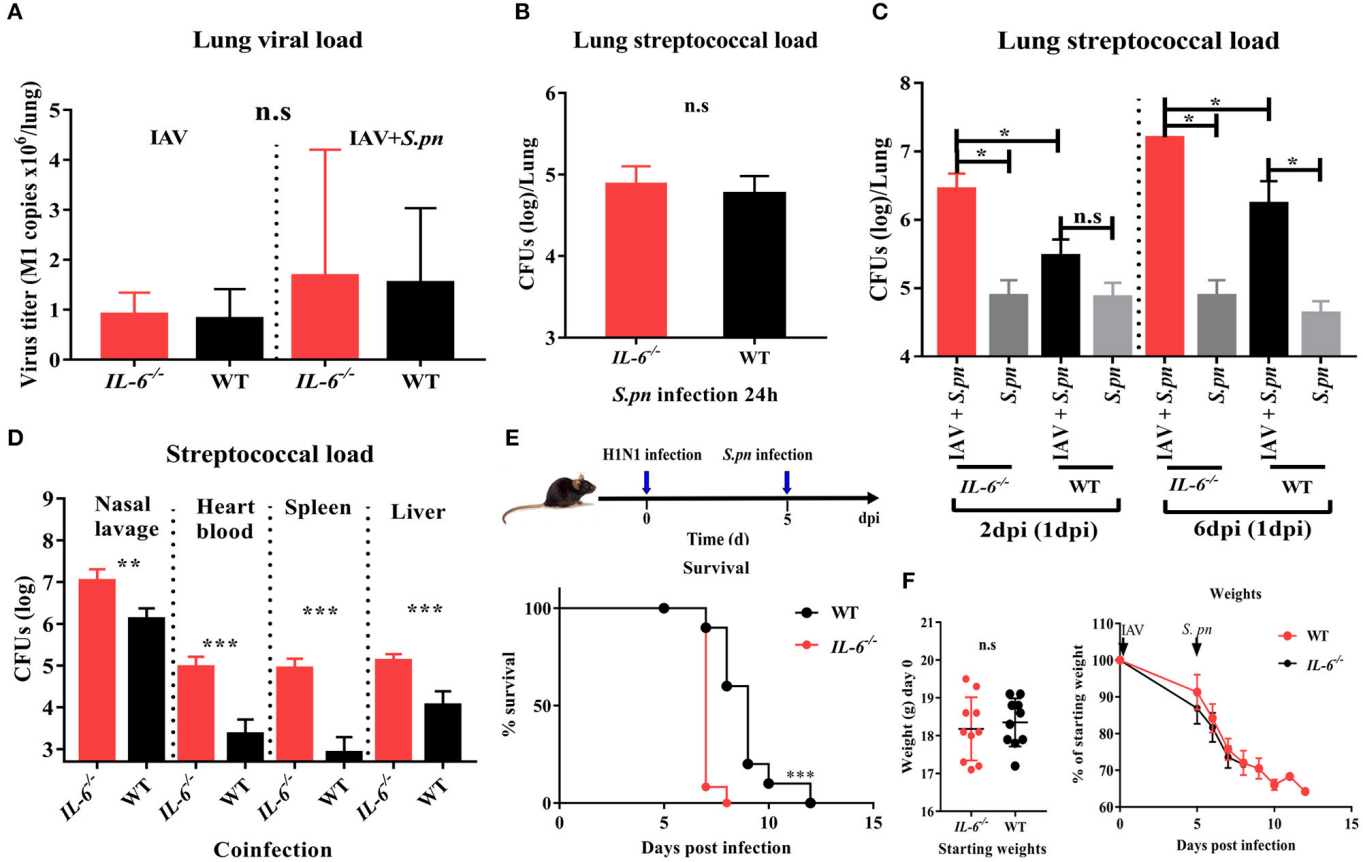
A deficiency in IL-6 increased susceptibility to secondary bacterial infection post influenza infection and decreased bacterial clearance. **(A)** Quantitative PCR for influenza matrix RNA in the lung during IAV infection and co-infection [6 dpi (1 dpi)] in *IL-6*^−/−^ and WT mice, *n* = 4–8/group. **(B)** Pneumococcal burden in the lung after single *S. pneumoniae* infection (24 h) in *IL-6*^−/−^ and WT mice, *n* = 8/group. **(C)** Pneumococcal burden in the lungs were evaluated at days 2 and 6 co-infection in *IL-6*^−/−^ and WT mice, *n* = 4–5/group. **(D)** Pneumococcal burden in nasal lavage, heart blood, spleen and liver of co-infected [6 dpi (1 dpi)] *IL-6*^−/−^ and WT mice, *n* = 5–10/group. **(E,F)** Mortality **(E)** and weight **(F)** following co-infection [6 dpi (1 dpi)] in *IL-6*^−/−^ and WT mice, *n* = 10/group. ^*^P < 0.05, ^**^P < 0.01, and ^***^P < 0.001.

To investigate if IL-6 influences the susceptibility of IAV-infected animals to secondary bacterial infection, we monitored viral titer and bacterial load in WT and *IL-6*^−/−^ mice. *IL-6*^−/−^ mutation was confirmed via PCR and PAGE (data not shown). We then evaluated the effects of this knock-out on the host's response to infection with IAV alone or *S. pneumoniae* alone. The viral titer showed that a lack of IL-6 didn't alter viral clearance in the lungs (Figure 4A). *IL-6*^−/−^ mice also had an equivalent bacterial burden when compared to WT mice post 24 h *S. pneumonia* infection (Figure 4B). It was previously verified that bacterial superinfection in WT animals was easiest post 6 days IAV infection, and most difficult post 2 days IAV infection (Figure 1C). However, bacterial superinfection in *IL-6*^−/−^ mice became easy post 2 days IAV infection (Figure 4C). The bacterial loads of the nasopharynx, heart blood, spleen, and liver showed deficiency in IL-6 led to decreased bacterial clearance and more severe disease spread (Figure 4D).

**Figure 5:**
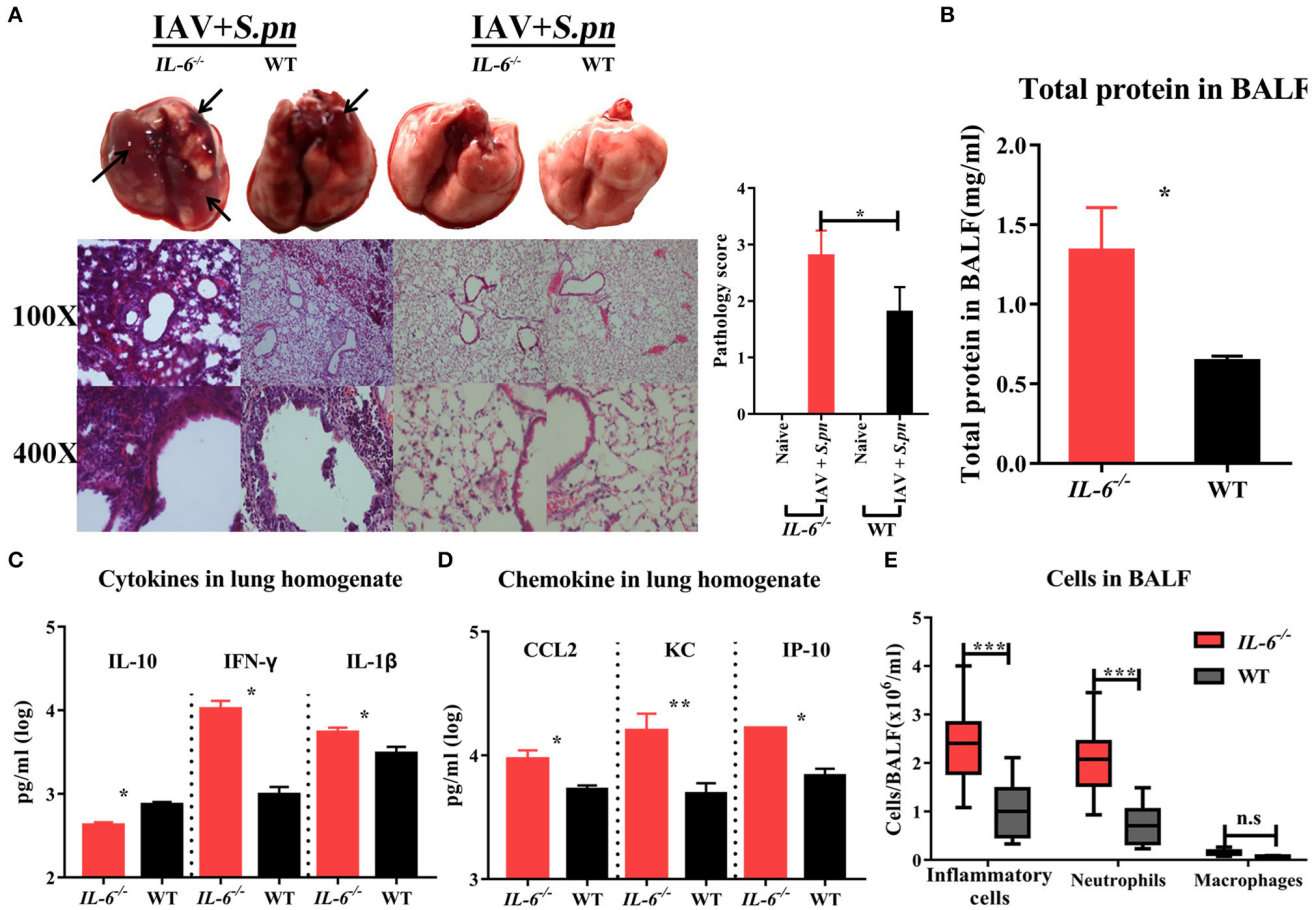
Influenza-*S. pneumoniae* co-infected pneumonia had higher cellular infiltration in *IL-6*^−/−^ mice than WT mice. **(A)** H&E staining of lungs during co-infection compared to naïve animals in *IL-6*^−/−^ and WT mice, *n* = 3/group. **(B)** Total protein in BALF from co-infected *IL-6*^−/−^ and WT mice, *n* = 5/group. **(C)** The cytokines (IL-10, IFN-γ, and IL-1β) in co-infected *IL-6*^−/−^ and WT mice, *n* = 10/group. **(D)** The chemokine (CCL2, KC, and IP-10) levels in co-infected *IL-6*^−/−^ and WT mice, *n* = 10/group. **(E)** Effects of IL-6 deficiency on lung inflammatory cells influx during co-infection. The total number of inflammatory cells was counted, and the total number of neutrophils and macrophages was determined, *n* = 8/group. All co-infected groups were established as 6 dpi (1 dpi). ^*^P < 0.05, ^**^P < 0.01, and ^***^P < 0.001.

To further investigate the effect of IL-6 on the mortality during co-infection, the survival rates of WT and *IL-6*^−/−^ mice was observed. Due to the synergistic effects of IAV and *S. pneumoniae*, both co-infected WT and *IL-6*^−/−^ mice developed 100% mortality and excessive weight loss (Figures 4E,F). In order to further explore the role of IL-6, pulmonary pathological changes including edema formation, cytokines and inflammatory cells from WT and *IL-6*^−/−^ mice were examined. Microscopic analysis of the pathological features of co-infection in the lungs from WT and *IL-6*^−/−^ mice showed large lesions; destruction of alveolar structures and vessels; inflammatory cells infiltration and degeneration, and shedding of airway epithelium (Figure 5A). Both macroscopic and microscopic pathological changes were more serious in *IL-6*^−/−^ mice (Figure 5A). IL-6 deficiency also resulted in increased barrier function failures when compared with WT mice (Figure 5B). IL-10 acts as an immunoregulator, inhibiting pro-inflammatory responses and prevents tissue damage (28). Increased IFN-γ signaling exacerbates bacterial pneumonia post-influenza infection; however, IL-6 has inhibition effects on IFN-γ (29, 30). In our severe model, IFN-γ and IL-1β were upregulated, while IL-10 was down-regulated during co-infection in *IL-6*^−/−^ mice (Figure 5C).

The first lines of host defense against pathogens are the phagocytes cells, including macrophages and neutrophils (31, 32). As CCL2, KC (IL-8) and IP-10 were all significantly up-regulated (Figure 5D), we sought to determine whether their high expression levels were accompanied by more pronounced macrophage and neutrophil accumulation in BALF of *IL-6*^−/−^ animals. IL-6 defects resulted in a marked increase in inflammatory cells (mainly neutrophils, approximately 86.3%) in the lungs after co-infection (Figure 5E, Figure S2). However, macrophage accumulation was not influenced by IL-6 expression in co-infected mice (Figure 5E, Figure S2).

To summarize, loss of IL-6 increased the susceptibility of bacterial superinfection after flu and weaken pathogen clearance, both which result in the early dissemination of bacteria in animals with co-infected pneumonia.

### IL-6 Played a Critical Role in Reducing Cells Death and Improving Macrophages Phagocytosis Function in Influenza- *S. pneumoniae* Co-Infected Pneumonia

**Figure 6:**
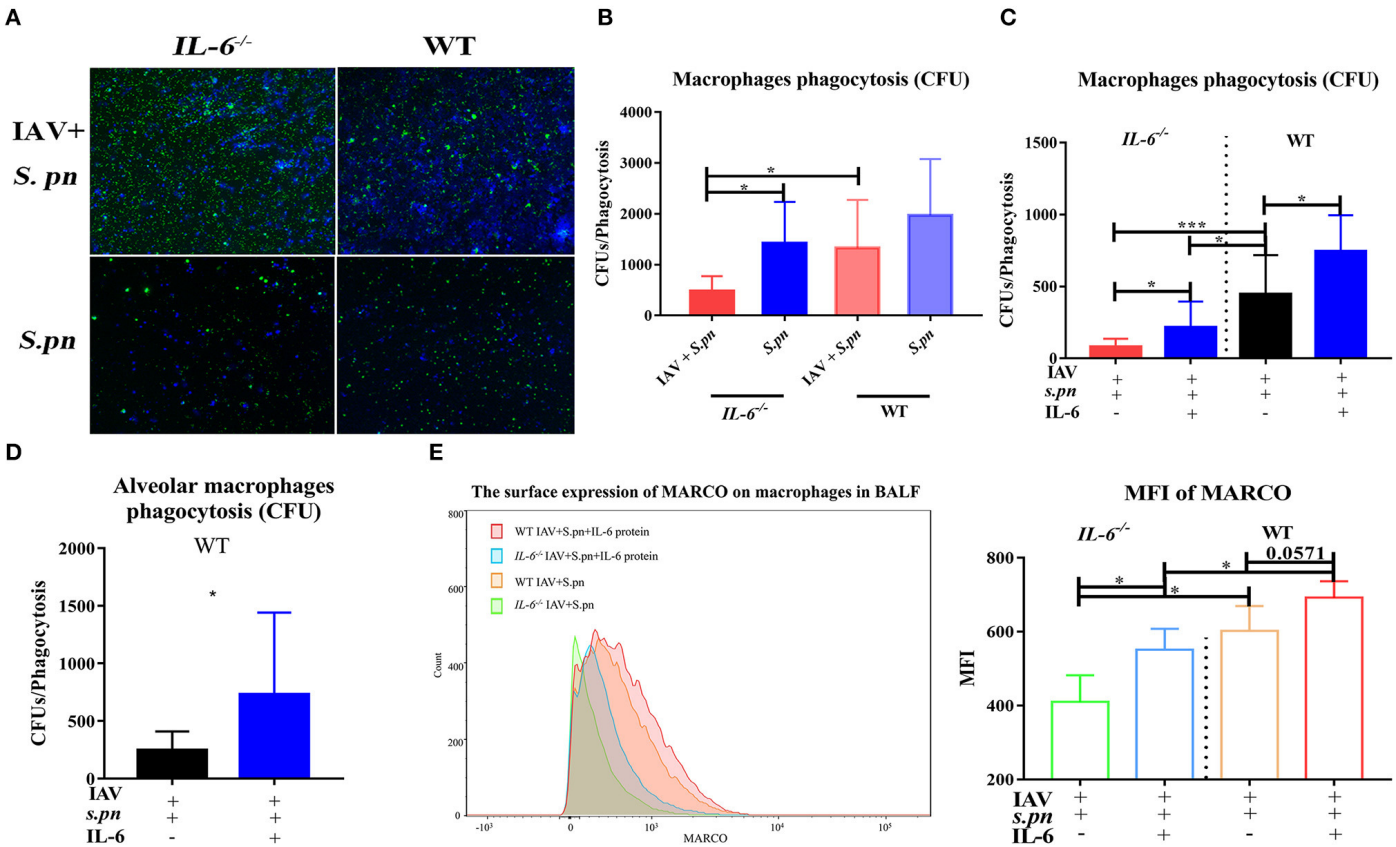
IL-6 improved macrophages phagocytosis function. **(A)** *In vivo* phagocytosis assays of co-infected *IL-6*^−/−^ and WT mice, analyzed and sorted by fluorescence, *n* = 4/group. **(B–D)** *In vitro* CFU-based assays of *S. pneumoniae* phagocytosis analysis of peritoneal macrophages **(B,C)** and alveolar macrophages **(D)** function, *n* = 6/group. **(C,D)** Macrophage phagocytosis activity was also evaluated in the presence or absence of IL-6 (20 ng/ml), *n* = 6/group. **(E)** Flow cytometry strategy for analyzing the surface expression of MARCO in macrophages in the BALF from co-infected *IL-6*^−/−^ and WT mice [6 dpi (1 dpi)] when intranasally treated with exogenous recombinant mouse IL-6 protein (10 μg/30 μl/mice), *n* = 5/group. ^*^P < 0.05 and ^***^P < 0.001.

To probe the reason for the increases in immune cells, bacterial load and apoptosis, we first detected cellular apoptosis using flow cytometry. Primary IAV infection promoted cellular apoptosis in the lung (12) (Figure S3) and IL-6 deficiency was not conducive to lung inflammatory cell survival, especially during co-infection (Figure S3). Moreover, the massive recruitment of inflammatory cells into co-infected lungs may help the body control bacteria, and impairment of their function may contribute to co-infection. We next examined the phagocytic capacity of these cells in the BALF using the *in vivo* phagocytosis assays. As expected, as a result of impaired phagocytosis, a large number of FITC-labeled bacteria can be directly visualized under the fluorescent microscope in phagocytes from co-infected *IL-6*^−/−^ mice (Figure 6A). To further determine the main type phagocytosis-impaired phagocytes in these animals, the phagocytic properties of both neutrophils and macrophages were evaluated. In influenza-infected macrophages from *IL-6*^−/−^ animals, the phagocytosis of *S. pneumoniae* was weakened (Figure 6B). However, there was no difference in neutrophils (data not shown). For further verification, we evaluated phagocytosis function of macrophages in the presence of exogenous recombinant IL-6 protein. Treatment with exogenous IL-6 protein significantly rescued the function of macrophages from *IL-6*^−/−^ mice and enhanced their function in WT mice (Figure 6C). We also repeated phagocytosis experiments using mouse alveolar macrophages and consistent result with peritoneal macrophages were showed (Figure 6D). The A scavenger receptor MARCO is responsible for binding and phagocytosis of *S. pneumoniae* (33). Therefore, we aimed to evaluate if there was a relationship between IL-6 and MARCO allowing for the phagocytosis of *S. pneumoniae*. Further analysis of the macrophage population showed that IL-6 treatment increased surface expression of MARCO in both WT and *IL-6*^−/−^ animals (Figure 6E), which was consistent with the CFU-based bacterial phagocytosis analysis.

It showed that IFN-γ produced in the lung after viral infection inhibited bacterial clearance through suppression of MARCO expression (34). Thereafter, we evaluated if IL-6 expression influenced MARCO expression through it mediated suppression on IFN-γ. Initially, we observed loss of IL-6 during co-infection resulted in higher levels of IFN-γ in the lung homogenate (Figure 5C) and BALF (Figure S4A) of mice. Last, exogenous IL-6 treatment significantly inhibited IFN-γ production in *IL-6*^−/−^ mice during co-infection (Figure S4A). All these certified that IL-6 could mediate suppression on IFN-γ. However, the expression of MARCO on macrophage surface didn't increase obviously after neutralization of IFN-γ in both *IL-6*^−/−^ and WT mice (Figures S4B,C). These results suggested that IL-6 may not depend on the inhibition of IFN-γ to increase surface expression of MARCO in macrophages during co-infection.

Taken together, these results showed that IL-6 not only affected the death of lung inflammatory cells but also improved macrophages phagocytic functions by increasing MARCO expression during co-infection.

### IL-6 Plays an Important Protection Role Allowing Animals to Resist Secondary Bacterial Infection Following IAV-Infection

**Figure 7:**
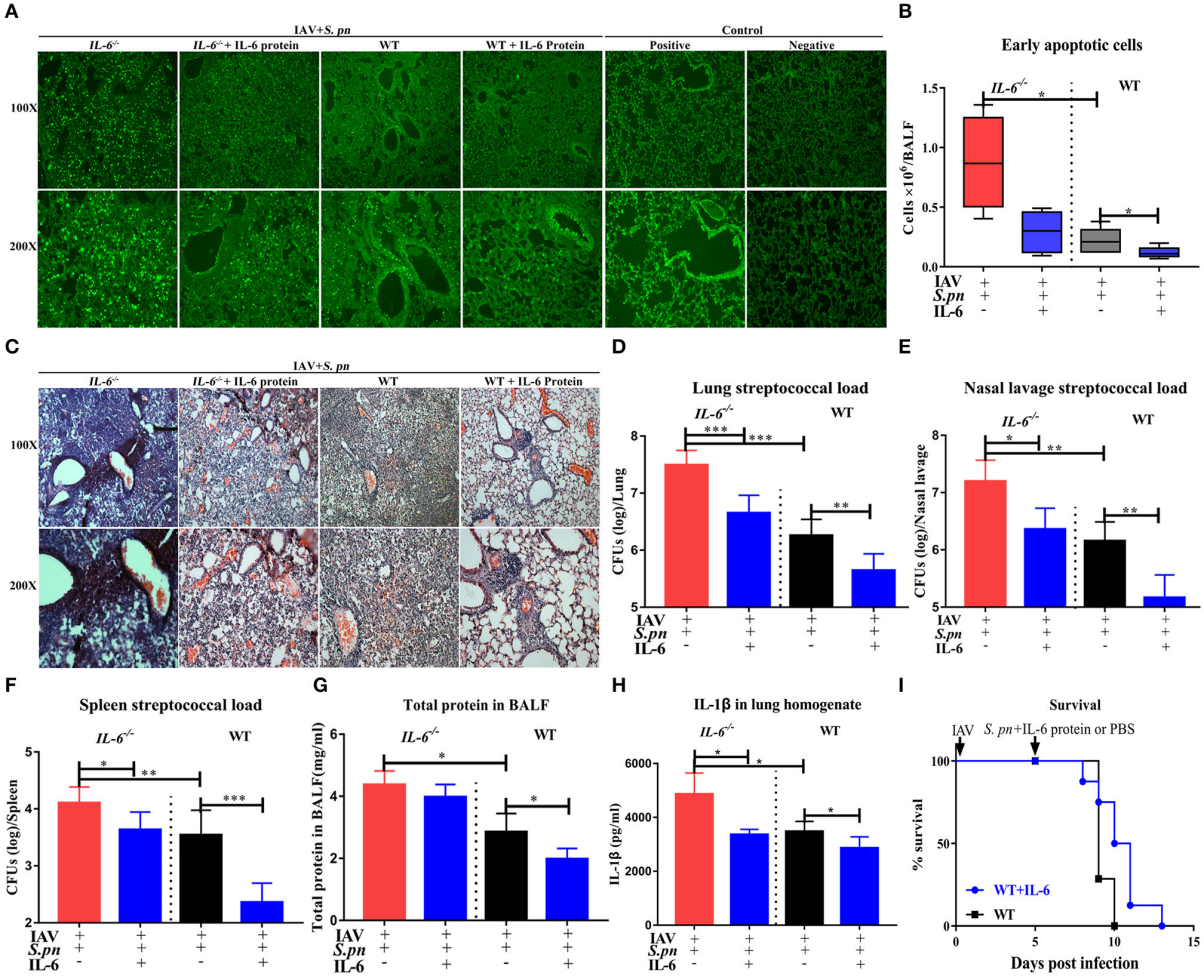
IL-6 reduced pulmonary cells death and promoted clearance of *S. pneumoniae* in co-infected animals. **(A)** Cellular apoptosis *in situ* of lung tissues during co-infection with or without IL-6 protein treatment, *n* = 3/group. **(B)** The early apoptotic cells in the BALF in co-infected *IL-6*^−/−^ and WT mice with or without IL-6 protein treatment, *n* = 5/group. **(C)** H&E staining of lung tissues during co-infection with or without IL-6 protein treatment, *n* = 3/group. **(D–F)** Pneumococcal burdens in pulmonary **(D)**, nasal lavage **(E)**, and spleen **(F)** tissues of co-infected mice when intranasally treated with exogenous recombinant mouse IL-6 protein, *n* = 7–13/group. **(G)** Total protein in the BALF from co-infected *IL-6*^−/−^ and WT mice with or without IL-6 protein treatment, *n* = 5/group. **(H)** The IL-1β level in the lungs of co-infected *IL-6*^−/−^ and WT mice with or without IL-6 protein treatment, *n* = 5/group. **(I)** The survival rates of co-infected WT mice with or without IL-6 protein treatment, *n* = 8/group. All mice were treated with IL-6 protein as followed: 10 μg/30 μl/mice of recombinant mouse IL-6 protein (R&D Systems) or PBS intranasal treatment at the time of *S. pneumoniae* challenge. All co-infected groups were established as 6 dpi (1 dpi). ^*^P < 0.05, ^**^P < 0.01, and ^***^P < 0.001.

To determine if the IL-6 protein can dramatically increase resistance to secondary bacterial infections, we used exogenous recombinant mouse IL-6 protein to rescue bacterial resistance in IAV-infected mice. As we expected, addition of exogenous IL-6 protein significantly reduced cells death in lung tissues (Figure 7A) and in BALF (Figure 7B). Pulmonary histology also revealed that exogenous IL-6 protein significantly extenuated lung inflammation and cells infiltration in both *IL-6*^−/−^ and WT mice (Figure 7C). For clearance of bacteria, treatment with IL-6 potently reduced pneumococcal burdens in lung, nasal lavage and spleen of infected mice when compared to WT mice treated with the PBS carrier (Figures 7D–F). Treatment with IL-6 also decreased the level of total protein (Figure 7G) and cytokines (Figure 7H). However, one IL-6 protein treatment didn't effectively change the survival of animals during fatal pneumonia infections as still 100% mortality (Figure 7I). Thus, treatment of influenza-infected WT mice with a single dose of IL-6 at the time of *S. pneumoniae* challenge is effective against secondary pneumococcal pneumonia, but can't effectively change the survival of co-infected mice.

**Figure 8:**
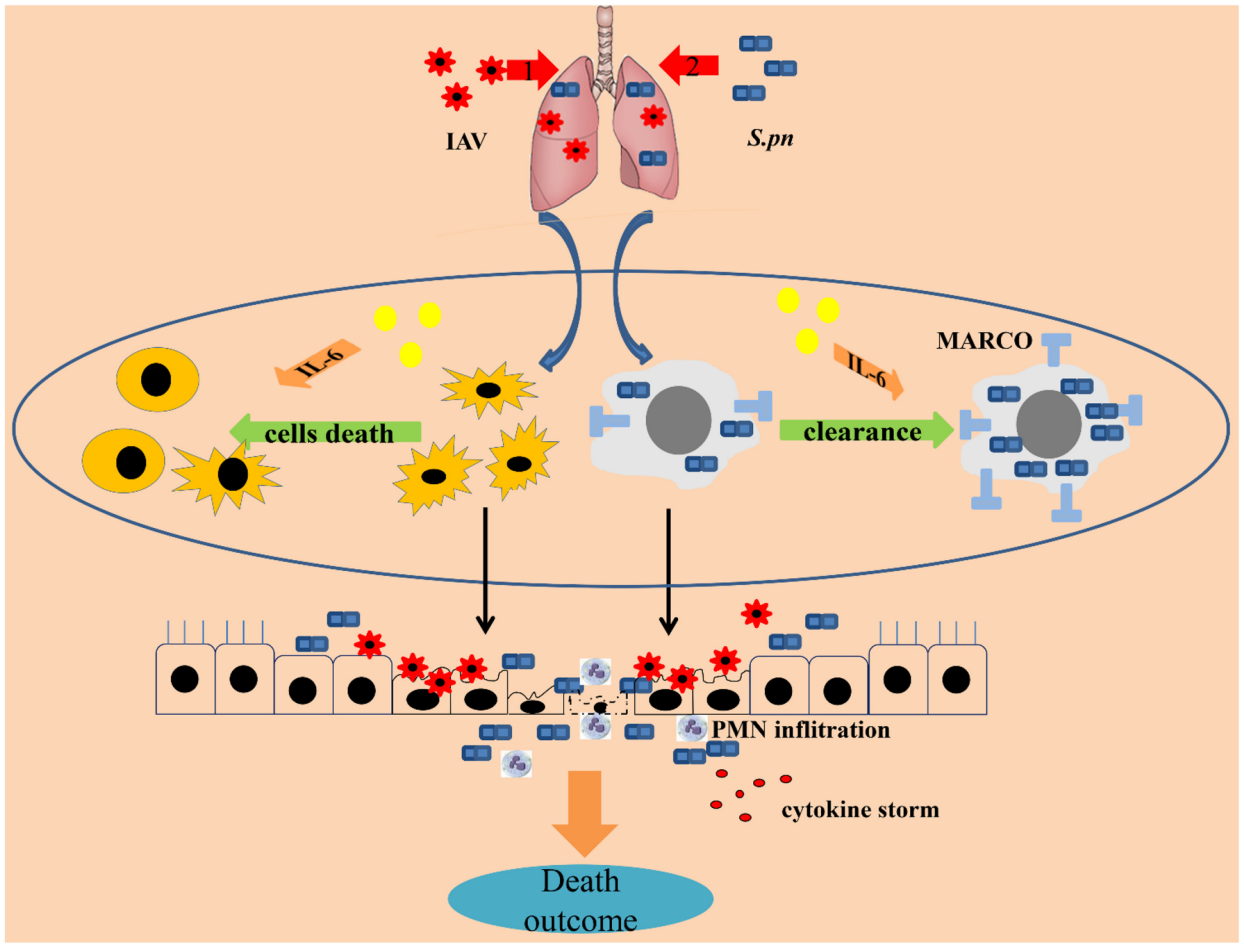
IL-6 protects co-infected animals. The protective characteristics of IL-6 during co-infected pneumonia were reduced cell survival and improved macrophage phagocytosis in the lungs. IL-6 rescued cells in the BALF from influenza-triggered apoptosis, maintaining homeostasis, one of the first lines of host defense against pathogens, leading to decreased susceptibility to secondary infection. In addition, IL-6 increased surface expression of MARCO in macrophages enhancing their capacity for phagocytosis during secondary bacterial infections. In conclusion, IL-6 acts as a cellular protectant during co-infection.

Together, this paper describes the protective functions of IL-6 during co-infected pneumonia. These predominantly reduce cells death and improve macrophage phagocytosis in the lungs. IL-6 reduced cells death in BALF from influenza-triggered death maintaining homeostasis, leading to decreased susceptibility to secondary infection. In addition, IL-6 mediated increased surface expression of MARCO on macrophage enhancing their phagocytic capacity during secondary bacterial infection (Figure 8).

## Discussion

This study evaluated whether IL-6 had an important role in resisting secondary bacterial infections post influenza infection. This study was able to show that IL-6 is crucial for cell death and macrophage phagocytosis enhancing resistance to superinfected *S. pneumoniae*, resulting in decreased susceptibility to secondary *S. pneumoniae* infection after primary influenza infection.

Having an adequate macrophage response is critical to an effective pulmonary host defense. When the bacterial burden exceeds the antimicrobial capacity of macrophages there is a critical failure in the immune response (2). As expected, IL-6 deficiency appeared to impair macrophage phagocytic function during co-infection (Figure 6B), while treatment with recombinant IL-6 significantly enhanced macrophage activity (Figures 6C,D). It has also been reported that IL-6 from peritoneal macrophages suppresses the uptake of oligomannose-coated liposomes partly through the inhibition of SIGNR1 expression (35). It has been reported that MARCO is responsible for the phagocytosis of pneumococci (33). However, there is still no report stipulating the relationship between IL-6 and MARCO. We found that impaired macrophage phagocytosis was accompanied by decreased MARCO expression during IL-6 deficiency (Figure 6E). And that treatment with recombinant IL-6 significantly increased MARCO expression (Figure 6E). IL-6 is known to be negative regulators of IFN-γ (29), in addition IFN-γ suppresses the expression of MARCO in alveolar macrophages inhibiting bacterial clearance in the lung after viral infection (34). So, we want to identify whether IL-6 regulates the expression of MARCO through IFN-γ. However, the results showed that the expression of MARCO on macrophage surface didn't increase obviously after neutralization of IFN-γ in both *IL-6*^−/−^ and WT mice (Figures S4B,C). It suggests that there are other factors regulating the expression of MARCO, which are regulated by IL-6. Further studies will be performed to imply related mechanism.

In our study, we also found that primary influenza infection and co-infection both cause apoptosis in the lung; however, the latter was more serious (Figure 5D). It has been reported that IL-6 signaling prevented CD133+ stem-like cells of lung cancer (36) and was essential for the resolution of H1N1 influenza infection by promoting neutrophils survival (14). Because ~86.3% of the total inflammatory cells in our study were neutrophils, we speculated that IL-6 may promote neutrophil survival; however, more experimentation will be necessary to validate these results. Neutrophil infiltrate was more obvious in IL-6 knockout mice than in WT animals (Figure S2). This is probably the result of a combination of decreased IL-6 and sIL-6R and increased activation of endothelial cells producing MCP-1 (16, 33).

The role of IL-6 in co-infection may be dependent on the influenza strain and the pathology of the virus. Previous studies have shown that IL-6 does not contribute to the pathogenesis of avian H5N1 influenza which is primarily mediated by a cytokine storm (37, 38). In addition, it does not seem to affect primary infection with a poorly pathogenic H3N2 strain that has minimal effect on lung function and host weight (20). In addition, *S. aureus, H. influenzae* and other often colonized bacteria in the upper respiratory tract are frequent secondary invaders post-influenza infection (3). In our studies, we didn't establish if IL-6 has a similar effect on secondary infection with these bacteria or other *S. pneumoniae* serotypes (39).

In conclusion, these findings demonstrate that IL-6 from the lungs induced by influenza infection exerts a protective effect on the host preventing secondary pneumococcal infections in the post-influenza period. Our results indicate that IL-6 resists secondary pneumococcal pneumonia in several different ways. IL-6–mediated enhancement of the antibacterial responses presents a unique direction for research in combating complicated influenza pneumonia and secondary bacterial infections. This protective immune mechanism will help us to understand the potential impact of immune components during complex infections and provide potential therapeutic options for influenza-*S. pneumoniae* co-infection.

## Data Availability Statement

The datasets generated for this study are available on request to the corresponding author.

## Ethics Statement

The studies involving human participants were reviewed and approved by the Clinical Research Ethics Committee of the Children's Hospital of Chongqing Medical University. Written informed consent to participate in this study was provided by the participants' legal guardian/next of kin. The animal study was reviewed and approved by the Institutional Animal Care and Use Committee of the Chongqing Medical University.

## Author Contributions

Study conceived and funding secured by XZ, YY, JY, and HW. XZ and XG designed the study and wrote the manuscript with contributions from all authors. Experiments performed by XG, XW, JX, JC, and SL and data analyzed by all authors.

### Conflict of Interest

The authors declare that the research was conducted in the absence of any commercial or financial relationships that could be construed as a potential conflict of interest.
