# Supplementary material for: IL-6 During Influenza-Streptococcus pneumoniae Co-Infected Pneumonia—A Protector
Source: Front Immunol. 2020 Jan 21;10:3102. doi: 10.3389/fimmu.2019.03102 (PMC6985362; doi:10.3389/fimmu.2019.03102)
Supplement: Supplementary file 1 [file Data_Sheet_1.pdf]

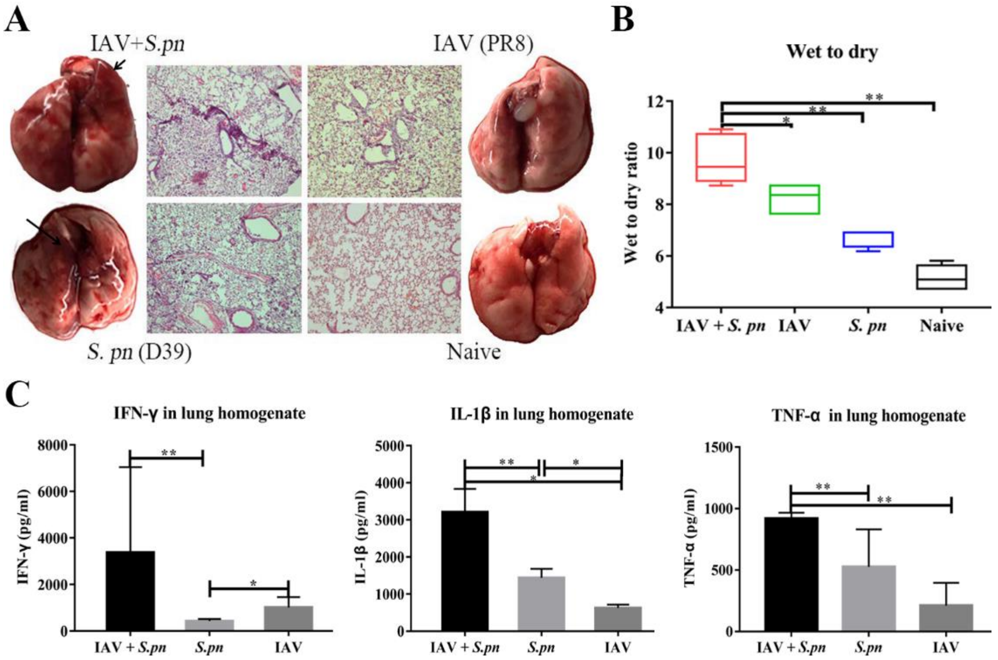

Figure S1. The characteristics of WT mice during severe co-infection. (A) Representative pathological analyses of the lungs of WT mice during co-infection or mock infection (naïve), single IAV infection, and single *S. pneumoniae* infection, n=3-5/group. (B) The wet/dry ratio of lungs was calculated to evaluate lung tissue edema, n=5/group. (C) Cytokines (IFN-γ, IL-1β and TNF-α) in the lungs of mice during co-infection and single infection, n=5. All co-infected groups were established as 6dpi (1dpi). *P<0.05, **P<0.01 and ***P<0.001.

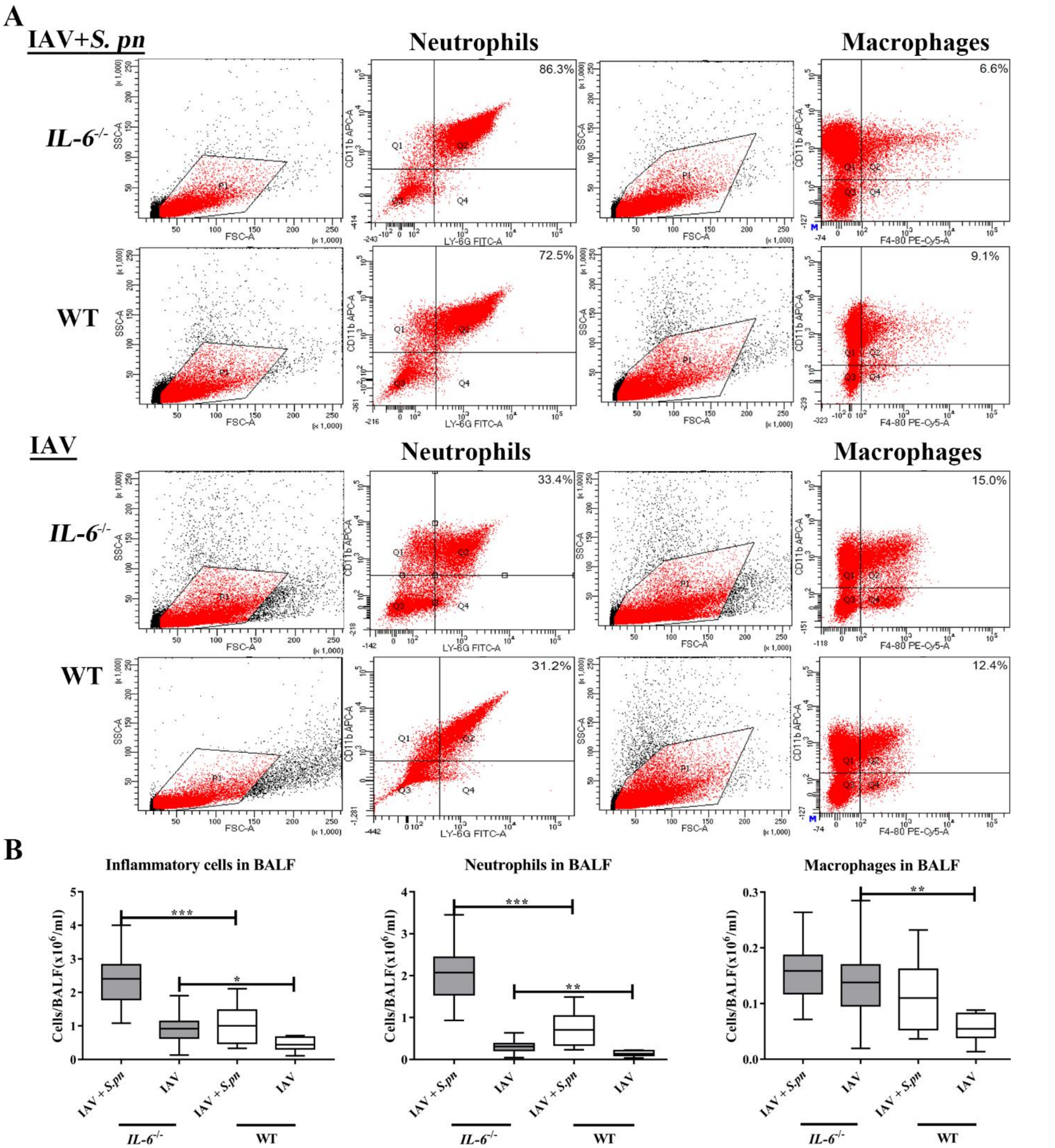

Figure S2. ***IL-6*$^{-/-}$ mice had higher cellular infiltration than WT mice during co-infection and IAV infection alone.** (A) Flow cytometry strategy for analyzing the percentage of neutrophils and macrophages in the BALF of co-infected and single IAV-infected *IL-6*$^{-/-}$ and WT mice, n=8/group. (B) The total number of inflammatory cells was counted, and the total number of neutrophils and macrophages was determined, n=8/group. All co-infected groups were established as 6dpi (1dpi). *P<0.05, **P<0.01 and ***P<0.001.

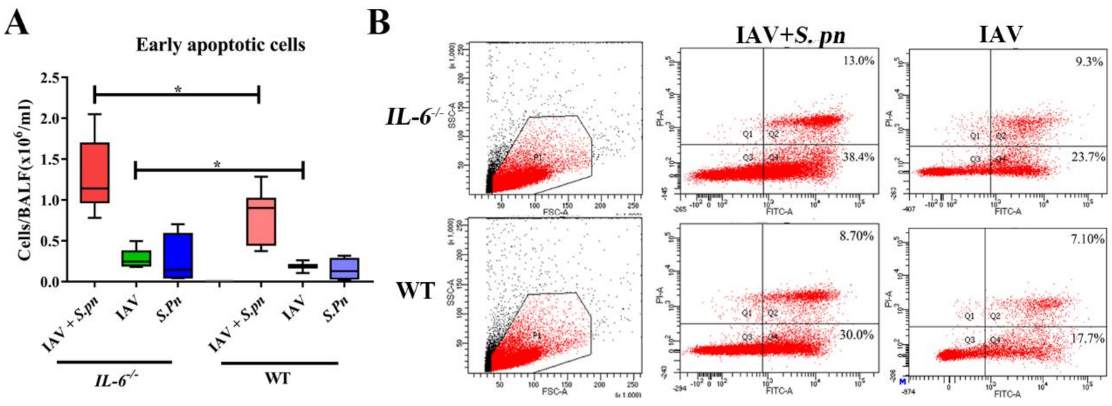

Figure S3. Influenza-*S. pneumoniae* co-infected pneumonia had higher cellular apoptosis in *IL-6*$^{-/-}$ mice than WT mice. (A) The early apoptotic cells in the BALF of *IL-6*$^{-/-}$ and WT mice during co-infection and IAV infection, n=7/group. (B) Flow cytometry strategy for analyzing the percentage of cellular apoptosis in the BALF of co-infected and IAV infected *IL-6*$^{-/-}$ and WT mice, n=8/group. All co-infected groups were established as 6dpi (1dpi). *P<0.05, **P<0.01 and ***P<0.001.

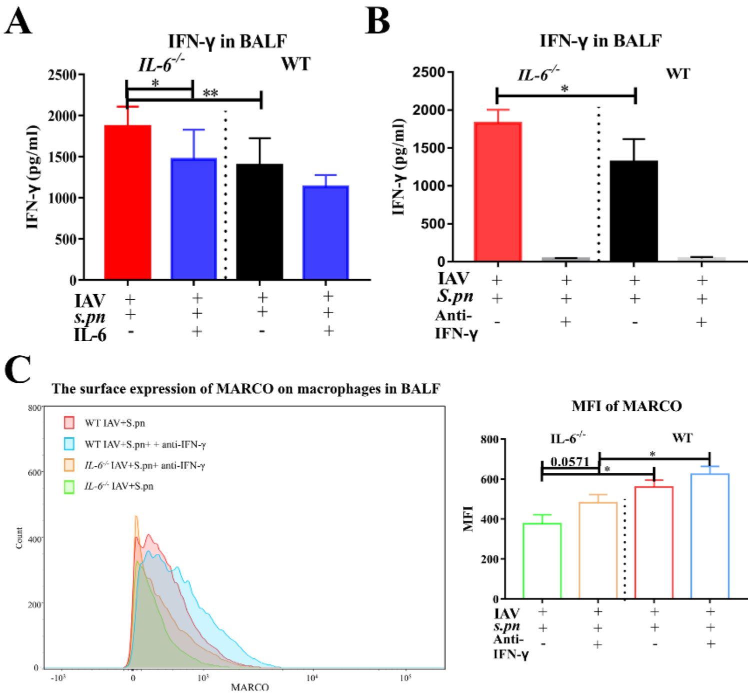

Figure S4. IL-6 increased expression of MARCO in macrophages partly through the inhibition of IFN-γ. (A) The level of IFN-γ in BALF from co-infected *IL-6*$^{-/-}$ and WT mice when intranasally treated with IL-6 protein, n=5/group. (B) The level of IFN-γ in BALF from co-infected *IL-6*$^{-/-}$ and WT mice when intranasally treated with IFN-γ neutralizing antibody, n=5/group. (C) Flow cytometry strategy for analyzing the surface expression of MARCO in macrophages in the BALF from co-infected *IL-6*$^{-/-}$ and WT mice when intranasally treated with IFN-γ neutralizing antibody, n=5/group. All co-infected groups were established as 6dpi (1dpi). *P<0.05, **P<0.01 and ***P<0.001.
